# Editorial: Cardiovascular disease prevention in the workplace

**DOI:** 10.3389/fcvm.2024.1473014

**Published:** 2024-09-06

**Authors:** Stefano Palermi, Josef Niebauer, Alessandro Biffi

**Affiliations:** ^1^ Public Health Department, University of Naples Federico II, Naples, Italy; ^2^ University Institute of Sports Medicine, Prevention and Rehabilitation, Paracelsus Medical University, Salzburg, Austria; ^3^ Med-Ex, Medicine & Exercise, Medical Partner Scuderia Ferrari, Maranello, Italy

**Keywords:** cardiovascular disease, cardiovascular prevention, corporate well-being programs, corporate wellness programs, corporate health check up

**Editorial on the Research Topic**
Cardiovascular disease prevention in the workplace

## Introduction

The European Society of Cardiology's current guidelines on cardiovascular (CV) disease prevention emphasize the need for a coordinated set of actions, including interventions in the workplace aimed at reducing or eliminating the impact of CV diseases and related disabilities (1, 2).

Although workplace health programs often target modifiable risk factors like nutrition, physical activity, and smoking cessation (3, 4), they are still rare, offer a narrow spectrum, and receive thus limited attention (2). This represents a big public health issue since company health and wellness interventions may provide an important opportunity to identify and manage CV risk (2). This oversight represents a significant public health concern as workplace health interventions provide critical opportunities to identify and manage CV risks. As employee demographics shift towards older age groups and the retirement age progressively extends, these preventive measures are set to become key components of both employee health maintenance and the strategic economic planning of companies. Also, companies are competing for the most talented and skilled employees, and attractive and comprehensive corporate health programs can serve as a pull-factor.

This Research Topic aimed to highlight the ideal features of a CV prevention program tailored to workplace environments.

## Analysis of included paper

Two studies within this topic focus on major CV risk factors in the workplace: stress and smoking habits.

The study by Fruscione et al. explore the profound effects of workplace stress on cardiovascular health, establishing a strong correlation between high-stress environments and elevated CV risk markers, corroborating findings from a recent meta-analysis (5). This study underscores the critical need for integrated stress management programs, suggesting interventions such as workplace yoga to mitigate these risks effectively (5). Costantino et al.’s cross-sectional analysis reveals an alarmingly high prevalence of smoking among employees, directly linking it to increased CV risk. The study advocates for comprehensive smoking cessation initiatives as essential components of corporate wellness strategies to substantially lower CV disease incidence as recommended by contemporary research (1).

Additionally, while not strictly focused on workplace settings, poor sleep and the COVID-19 pandemic are also identified as significant contributors to CV disease.

Hou et al. address the often-overlooked link between poor sleep quality and cardiovascular health, suggesting that disturbed sleep significantly elevates CV disease risk. Their research supports integrating sleep management into CV disease prevention strategies in the workplace, identifying sleep improvement as a crucial factor in risk mitigation (1). Niebauer et al. assess the impact of COVID-19 on CV health, noting both direct effects from the virus and indirect consequences such as increased stress and reduced physical activity during lockdowns (6, 7). The study highlights the need for adaptable physical activity programs to address the ongoing pandemic while supporting cardiovascular health, aligning with findings on the benefits of exercise for long COVID-19 recovery (8).

The concluding studies propose effective solutions for creating an ideal corporate health program centered around physical activity (9).

Amatori et al. provide robust evidence supporting the role of regular physical activity in mitigating CV risk among employees. They advocate for structured exercise programs that are adaptable to employee schedules, demonstrating that even moderate physical activity enhancements can significantly benefit heart health and overall well-being. This aligns with systematic reviews showing that physical activity not only benefits employees but also offers substantial economic returns for companies (10). Cristi Montero et al.’s research introduces “active breaks”—short, structured physical activity sessions during the workday. This innovative approach helps combat the sedentary nature of modern office environments and significantly reduces CV risk factors such as hypertension and obesity among employees.

## Conclusion

The collective research presented underscores the effectiveness of comprehensive CV disease prevention programs in the workplace. By addressing both direct risk factors like smoking and indirect factors such as stress, poor sleep and physical inactivity, alongside innovative solutions like active breaks and customized physical activity regimens, companies can significantly enhance employee health and productivity. As workplaces evolve, the integration of these holistic wellness strategies will be crucial in reducing the growing burden of CV disease among an aging workforce.
